# In Vitro and In Silico Analysis of the Anticancer Effects of Eurycomanone and Eurycomalactone from *Eurycoma longifolia*

**DOI:** 10.3390/plants12152827

**Published:** 2023-07-31

**Authors:** Nurhanan Murni Yunos, Habibah A. Wahab, Mohammad G. Al-Thiabat, Nor Jannah Sallehudin, Muhamad Haffiz Jauri

**Affiliations:** 1 Natural Products Division, Forest Research Institute Malaysia, Kepong 52109, Selangor, Malaysia; norjannah@frim.gov.my (N.J.S.); haffiz@frim.gov.my (M.H.J.); 2 School of Pharmaceutical Sciences, Universiti Sains Malaysia, Gelugor 11800, Penang, Malaysia; mohd.althiabat@gmail.com

**Keywords:** *Eurycoma longifolia*, quassinoids, cancer cell lines, apoptosis, TNF-α, DHFR, molecular docking, ADMET

## Abstract

Eurycomanone and eurycomalactone are known quassinoids present in the roots and stems of *Eurycoma longifolia*. These compounds had been reported to have cytotoxic effects, however, their mechanism of action in a few cancer cell lines have yet to be elucidated. This study was aimed at investigating the anticancer effects and mechanisms of action of eurycomanone and eurycomalactone in cervical (HeLa), colorectal (HT29) and ovarian (A2780) cancer cell lines via Sulforhodamine B assay. Their mechanism of cell death was evaluated based on Hoechst 33342 assay and in silico molecular docking toward DHFR and TNF-α as putative protein targets. Eurycomanone and eurycomalactone exhibited in vitro anticancer effects manifesting IC_50_ values of 4.58 ± 0.090 µM and 1.60 ± 0.12 µM (HeLa), 1.22 ± 0.11 µM and 2.21 ± 0.049 µM (HT-29), and 1.37 ± 0.13 µM and 2.46 ± 0.081 µM (A2780), respectively. They induced apoptotic cancer cell death in dose- and time-dependent manners. Both eurycomanone and eurycomalactone were also predicted to have good inhibitory potential as demonstrated by the docking into TNF-α with binding affinity of −8.83 and −7.51 kcal/mol, respectively, as well as into DHFR with binding affinity results of −8.05 and −8.87 kcal/mol, respectively. These results support the evidence of eurycomanone and eurycomalactone as anticancer agents via apoptotic cell death mechanism that could be associated with TNF-α and DHFR inhibition as among possible protein targets.

## 1. Introduction

Cancer is the second leading cause of morbidity and mortality globally, with colorectal, cervical and ovarian cancers among the top leading causes of cancer-related deaths worldwide [1]. Cancer death rates have continued to rise globally from 7.6 million deaths in 2005 to 10 million deaths in 2020, which is nearly one in six deaths as reported by the WHO [2]. Many types of cancers are asymptomatic during their early stages, which can make them difficult to detect until they have advanced to a later stage. Late detection of these cancers normally leads to poor prognosis after treatments in many cancer patients. The main modality of treatment for treating advanced cancer is chemotherapy [3]. Currently, 90% of failures in chemotherapy are during the invasion and metastasis of cancers related to drug resistance [4] and due to various toxicity effects [5]. Due to cancer’s heterogeneity there are more than 100 types, [6] yet no single treatment or drug can treat all these cancers; thus, different drugs and treatment combinations are needed. Thus, the worldwide search for new anticancer agents to overcome these issues is ongoing.

One of the main factors that contribute to drug resistance is the dysregulation of apoptosis or programmed cell death [7]. Apoptosis plays a critical role in removing damaged or abnormal cells from the body, preventing them from dividing and growing uncontrollably by committing cell suicide. Apoptosis involves regulation and dysregulation of certain genes and proteins and these proteins are often classified as proapoptotic and oncoproteins, respectively. However, when apoptosis malfunctions, these damaged or abnormal cells may continue to divide and multiply, leading to the development of tumor or cancer [8,9,10]. One of the strategies for finding new anticancer drug candidates is to search for compounds that reinduce apoptosis and target key proteins/genes in cancer cells that can reinduce the mechanism of apoptosis.

Among the proteins directly involved in apoptosis include death receptors, such as tumor necrosis factor alpha (TNF-α), Fas ligand, the caspases and Bcl-2 family proteins, among others. TNF-α is a multifunctional cytokine that plays a key role in cell proliferation, metabolism, differentiation and survival [11,12,13,14,15]. It has also been identified as a potential therapeutic target for cancer treatment due to its ability to inhibit cancer development by inducing apoptosis [11,12,13,14,15]. In addition, there are also many proteins that are indirectly involved in the regulation of apoptosis but are also important in cell growth and proliferation, such as dihydrofolate reductase (DHFR), heat shock proteins (HSPs), kinases and phosphatases, and signal transduction proteins such as Ras and Raf. DHFR is one of the key proteins involved in the folate metabolism pathway that indirectly leads to apoptosis [16]. Folate metabolism is one of the well-known targets for cancer chemotherapy due to its role in nucleic acid synthesis [17,18,19]. It catalyzes the reduction of folate to tetrahydrofolate and maintains reduced cellular folate pools [17,18,19]. Inhibition of the DHFR enzyme reduces the amount of tetrahydrofolate that is required for the synthesis of pyrimidines and purines, which are necessary for one-carbon metabolism reactions [20,21,22]. As a result, inhibition of DHFR can halt the synthesis of RNA and DNA, leading to the death of cancer cells via apoptosis [16,20,21,22]. It is therefore suggested that DHFR and TNF-α are among attractive targets for the search and development of anticancer agents based on the apoptotic cell death mechanism [11,18,23].

Natural products, particularly those derived from plants, continue to be important sources of developing new anticancer agents and it was estimated that 64.9% of either natural products, or synthetic variations from novel structures of natural products, had been developed into cancer drugs [24]. *Eurycoma longifolia* is a popular herbal plant species belonging to the Simaroubaceae family that is widely distributed in Southeast Asian countries. *E. longifolia*, especially its roots, had also been reported to have various medicinal properties including antibacterial, cytotoxic/anticancer, antimalarial, antiulcer, antiparasitic and antipyretic [25]. The major compounds found in the roots of *E. longifolia* belonging to quassinoids (degraded triterpenes) are among the compounds known to contribute to various medicinal effects based on in vitro or/and in vivo studies including anticancer properties. As reviewed by Nurhanan et al. [26], quassinoids such as eurycomanone were reported to have in vitro anticancer effects against breast cancer, colon adenocarcinoma, fibrosarcoma, lung cancer cells and melanoma whereas eurycomalactone was reported to have in vitro anticancer effects against murine lymphocytic leukemia, epidermoid, melanoma, breast, lung and colon cancer cells. Despite their reported anticancer effects, the mechanism of action of these compounds in several cancer cell lines such as ovary, colorectal and cervical has not been fully elucidated. It was reported that 68 quassinoid analogues were designed and docked against DHFR via molecular docking and the results had shown their inhibitory effects against DHFR [27]. Other quassinoids, such as bruceantin and brusatol, were also found to inhibit DHFR in P388 leukemia cells [28]. In another study, brusatol was also reported to inhibit TNF-α [29]. In our previous proteomic analysis, we found that TNF-α was involved in apoptosis when treated with 9-methoxycanthin-6-one, another compound that was also isolated from *E. longifolia* [26]. DHFR and TNF-α are also known to be involved in apoptosis when cancer cells were treated with the known chemotheraphy drug, methotrexate [30,31]. However, to the best of our knowledge, no studies have been conducted to evaluate the inhibitory effects of eurycomanone and eurycomalactone against TNF-α and DHFR proteins. Hence, the aim of this study was to evaluate the in vitro anticancer effects and the mechanisms of action of eurycomanone and eurycomalactone via apoptosis as well as to evaluate the interactions of both compounds with DHFR and TNF-α and their drug-likeness activities via in silico studies.

## 2. Results and Discussion

### 2.1. Isolation and Characterization of Eurycomalactone and Eurycomanone

Eurycomanone (**1**) was isolated from *E. longifolia* as a white powder. Its molecular formula C_20_H_24_O_9_ was established by positive-ion HREIMS [M+H]^+^409.3851, (calcd C_20_H_24_O_9_ for *m*/*z* 408.403). Comparing its ^1^H and ^13^C-NMR spectra (Table 1) with those of (**2**), we found the chemical shift of methyl protons for 4 and 8-CH_3_ was increased to δ_H_ 1.81 and 2.00, respectively. The presence of *ortho* protons in C-14 and C-15 were displayed δ_H_ 3.25 and δ_H_ 5.25, respectively. Twenty signals were displayed in its ^13^C-NMR spectrum, including two carbonyl carbons at δ_C_ 197.89 (C-2) and δ_C_ 174.35 and four methylene carbons at δ_C_ 162.98 (C-3), 126.48 (C-4), 119.80 (C-13) and 108.71 (C-13’). 

Eurycomalactone (**2**) was a white powder and its molecular formula was determined as C_19_H_24_O_6_ by HREIMS at m/z 349.1647 [M+H]^+^, ^1^H and ^13^C-NMR spectra. The ^1^H-NMR spectrum of (**1**) showed signals at δ_H_ 6.13 (1H, s) corresponding to vinyl proton at C-3. Four signals resolved at δ_H_ 1.64, 1.96, 1.27 and 1.18 (3H, s) indicated methyl protons in compound (**1**). The ^13^C NMR peak assignments showed four methyl carbons resonated at δ_C_ 23.64, 21.97, 12.19 and 32.33. Meanwhile signals at δ 176.31, 197.41 and 205.56 were attributed to carbonyl carbon (C-2, C-3 and C-15).

**Figure 1:**
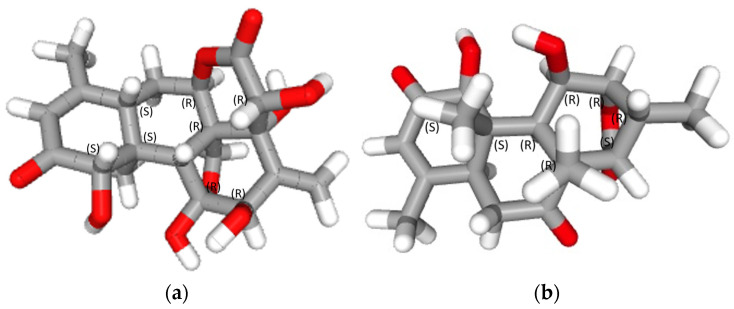
3D structures of (**a**) eurycomanone and (**b**) eurycomalactone with labeled chiral centers (S is left configuration and R is right configuration).

The known constituents (**1**) and (**2**) were identified by comparison of their spectral data ^1^H and ^13^C NMR and MS with those reported in the literature [32,33,34]. The structures of eurycomanone (**1**) and eurycomalactone (**2**) are shown in Figure 1.

### 2.2. Percentage of Cells Viability and IC_50_ Values

**Figure 2:**
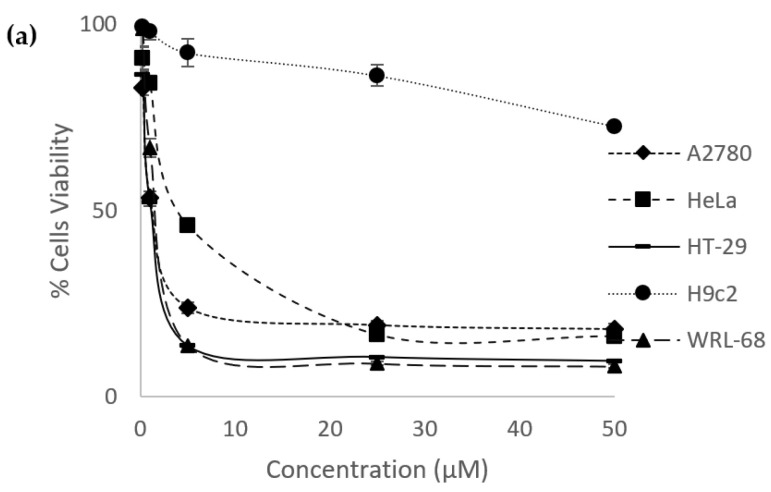

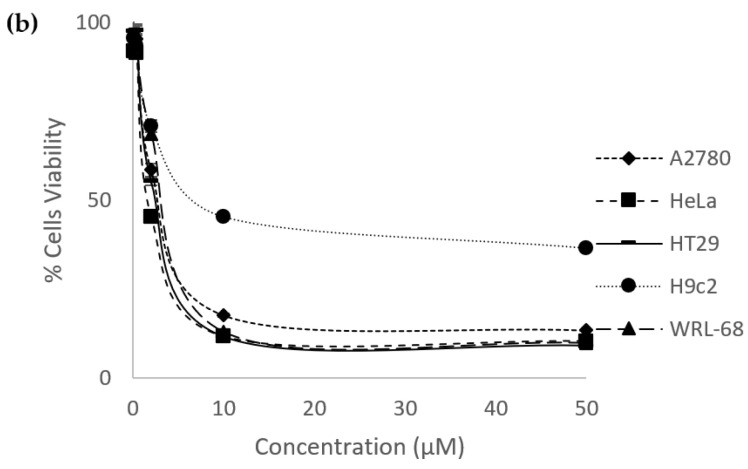
Dose–response curves of percentage of cells viability versus concentrations of (**a**) eurycomanone and (**b**) eurycomalactone when tested against ovarian (A2780), cervical (HeLa) and colorectal cancer (HT29) cancer cell lines and cardiomyocyte (H9C2) and liver (WRL-68) normal cell lines.

The in vitro anticancer effects of eurycomanone and eurycomalactone were evaluated against ovarian (A2780), cervical (HeLa) and colorectal (HT29) cancer cells, as well as normal cardiomyocyte (H9C2) and liver (WRL-8) cell lines. Cisplatin and methotrexate were also evaluated on their in vitro anticancer effects for comparison studies. Both drugs had been reported to treat various type of cancers, including ovarian, cervical and colorectal cancers [35,36,37]. Both eurycomanone and eurycomalactone gave significant in vitro anticancer effects, with IC_50_ values ranging from 1.22 ± 0.11 µM to 4.58 ± 0.090 µM (for eurycomanone) and 1.60 ± 0.12 µM to 2.46 ± 0.081 µM (for eurycomalactone), as illustrated in Figure 2 and tabulated in Table 2. Cisplatin exerted comparable in vitro anticancer activities with the range of IC_50_ between 1.38 ± 0.037 µM to 1.77 ± 0.018 µM, whereas, methotrexate exerted better in vitro anticancer activities with the range of IC_50_ between 0.016 ± 0.00050 µM and 0.094 ± 0.0043 µM, respectively (Figure 2, Table 2). Eurycomanone, eurycomalactone and methotrexate showed less cytotoxic effects in the cardiomyocyte H9c2 normal cell line as opposed to the liver WRL-68 normal cell line (Table 2). Clinically, cisplatin was reported to have several side effects, including cardiotoxicity, nephrotoxicity and neurotoxicity [5], whereas methotrexate was also reported to have adverse effects (e.g., hepatotoxicity, nephrotoxicity, gastrointestinal toxicity and death due to infections and hemorrhage) when given to cancer patients [38].

From our current studies, the in vitro anticancer analysis showed that eurycomanone and eurycomalactone were in the potent range since the IC_50_ values were less than 50 µM [39]. To the best of our knowledge, this is the first report that revealed eurycomalactone had in vitro anticancer effects against ovarian cancer cells. Other studies had also reported that eurycomanone and eurycomalactone gave in vitro anticancer effects against other cancer cell lines. Eurycomanone was reported to have anticancer effects in breast [40,41], colon, fibrosarcoma, lung and melanoma cancer cell lines [40], with IC_50_ values ranging from 0.49 to 35 µM, whereas, eurycomalactone was reported to have anticancer effects in murine lymphocytic leukemia (P388) and epidermoid (KB) [42], lung cancer (A-549), breast cancer (MCF-7) [41] and colon (26-L5), melanoma (B16-BL6) and lung (LLC and A549) cancer cell lines [43], with IC_50_ values ranging from 0.57 to 23.25 µM. However, information on the mechanisms of action of eurycomanone and eurycomalactone in killing the cancer cells is still lacking.

### 2.3. Apoptotic Effects of Eurycomanone and Eurycomalactone via Hoechst 33342 Assay

Apoptotic effects of eurycomanone and eurycomalactone against A2780, HT-29 and HeLa cancer cell lines were analyzed after performing Hoechst 33342 assay. These cell lines were also treated with these compounds at different concentrations and incubation times to preliminarily evaluate their pharmacodynamic effects in inducing apoptosis. Apoptosis is a programmed cell death in which the cells undergo distinct changes in their morphology, which normally start with chromatin condensation, DNA fragmentation, cell shrinkage, cell membrane blebbing and, finally, formation of apoptotic bodies. Hoechst assay is commonly used to stain the apoptotic cells in fluorescence blue [44].

**Figure 3:**
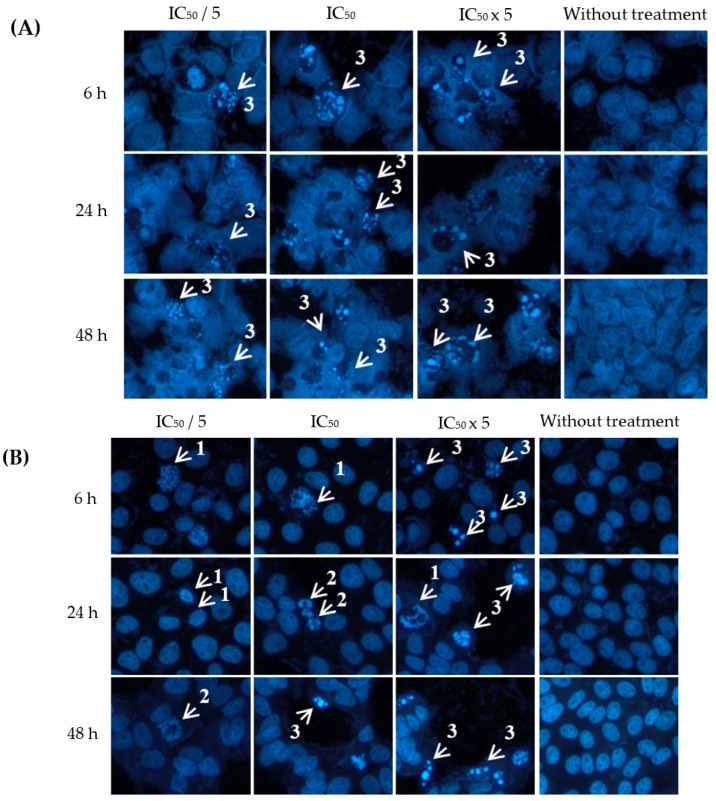

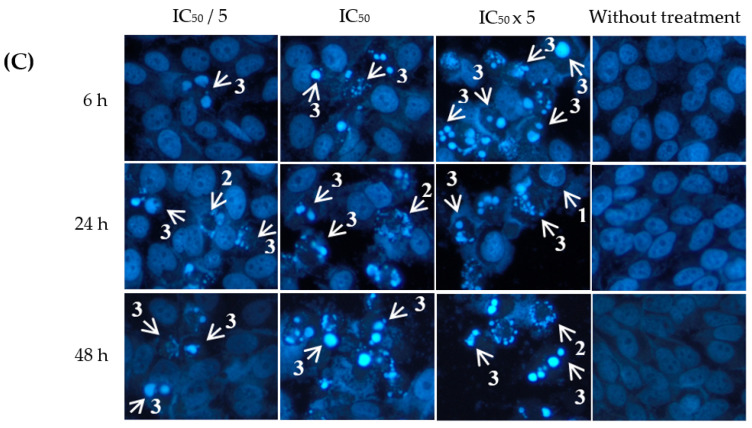
Visualization of (**A**) ovarian (A2780), (**B**) colorectal (HT29) and (**C**) cervical (HeLa) cancer cells when treated with eurycomanone at different concentrations (IC_50_/5, IC_50_, IC_50_x 5) and incubation times (6, 24 and 48 h) with Hoechst 33342 staining. Arrows indicate different stages of apoptosis: (1) extensive chromatin condensation, (2) highly compact chromatin at the periphery of the nucleus and (3) apoptotic bodies. Magnification: ×200.

As shown in Figure 3, Hoechst dye stained the occurrence of chromatin condensation, dense chromatin at the periphery of the nucleus and apoptotic bodies upon treatment with eurycomanone, whereas nonapoptotic cells had spherical nuclei and evenly distributed chromatin. The percentage of apoptotic indices are shown in Table 3. Eurycomanone significantly increased the percentage of apoptotic index in A2780, HT-29 and HeLa cells in a concentration- and time-dependent manner, especially at 24 h and 48 h incubation times and at the highest concentration of eurycomanone tested.

**Figure 4:**
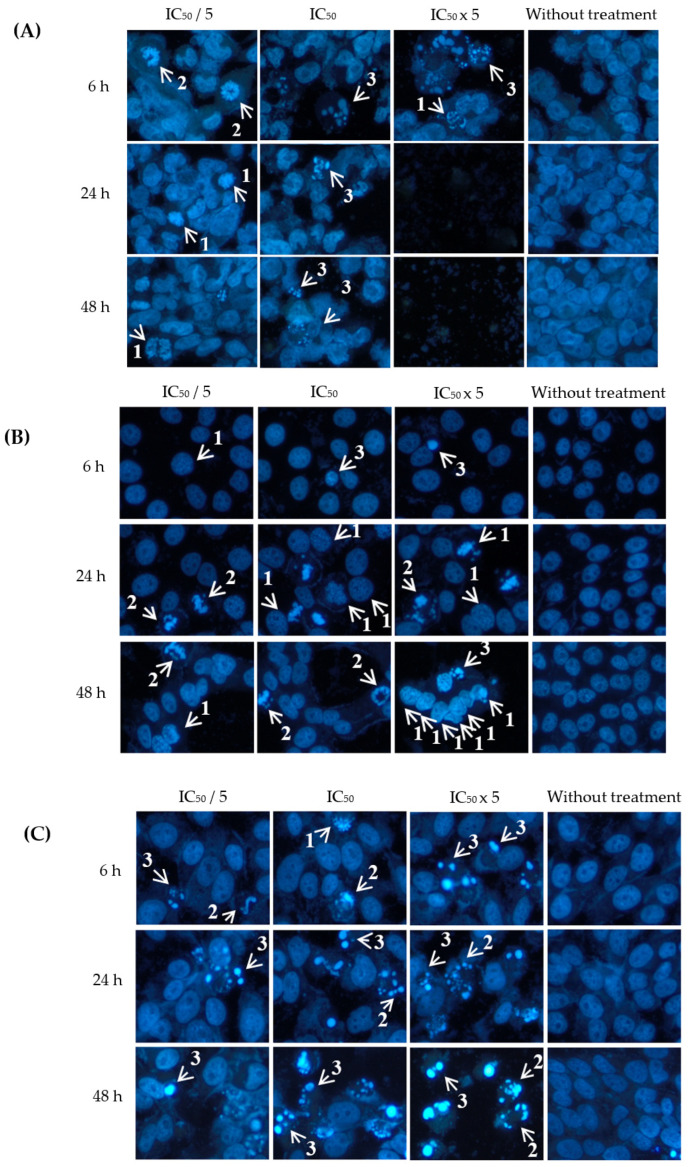
Visualization of (**A**) ovarian (A2780), (**B**) colorectal (HT29) and (**C**) cervical (HeLa) cancer cells when treated with eurycomanone at different concentrations (IC_50_/5, IC_50_, IC_50_x 5) and incubation times (6, 24 and 48 h) with Hoechst 33342 staining. Arrows indicate different stages of apoptosis: (1) extensive chromatin condensation, (2) highly compact chromatin at the periphery of the nucleus and (3) apoptotic bodies. Magnification: ×200.

As shown in Figure 4, eurycomalactone also induced apoptosis A2780, HT-29 and HeLa cells, in which different stages and hallmark signs of apoptosis were clearly shown, such as chromatin condensation, dense chromatin at the periphery of the nucleus and formation of apoptotic bodies. The percentage of apoptotic indices are shown in Table 4. Eurycomalactone significantly increased the percentage of apoptotic index in HT-29 and HeLa cells in a concentration- and time-dependent manner, especially at the highest concentration of eurycomalactone tested.

### 2.4. Molecular Docking Analysis

**Figure 5:**
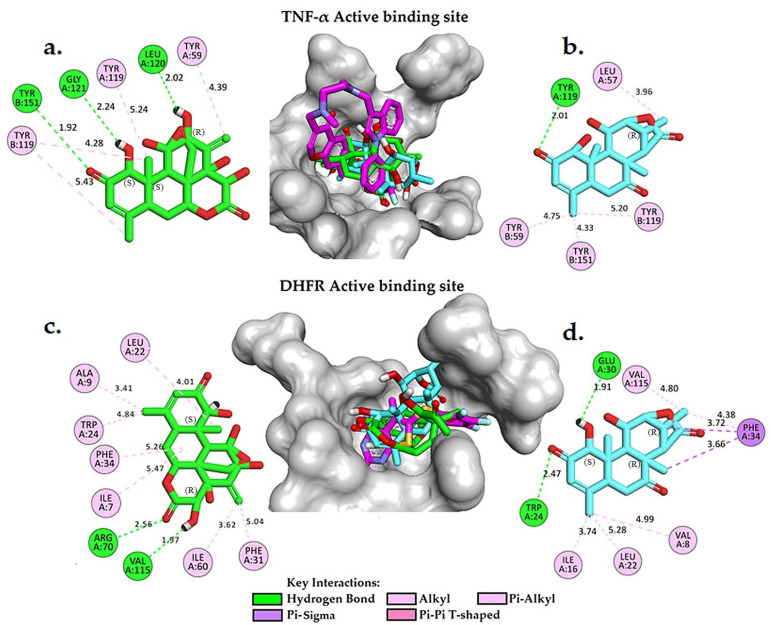
A 3D and 2D interaction analysis of docked models of eurycomanone (**a**) and eurycomalactone (**b**) with the TNF-α active binding site, and eurycomanone (**c**) and eurycomalactone (**d**) with the DHFR active binding site, was conducted using BIOVIA Discovery Studio Visualizer. The original crystal structure is depicted in pink for carbon (C), red for oxygen (O), cyan for fluorine (F), orange for sulphur (S) and blue for nitrogen (N). Eurycomanone is shown in green for C and red for O, and eurycomalactone is depicted in cyan for C and red for O.

Molecular docking is a widely used computational technique in modern drug design for predicting drug–receptor interactions and identifying potential inhibitors. In this study, we conducted a comparative analysis of the free binding energy and binding interactions of eurycomanone and eurycomalactone with the cocrystallised ligands in the active binding sites of TNF-α (PDB ID: 2AZ5) and DHFR (PDB ID: 5HQY) (refer to Table 5, Supplementary Figure S1 and Figure 5). Our aim was to gain insights into the potential mechanisms of action of eurycomanone and eurycomalactone against TNF-α and DHFR. To validate the docking process, we first redocked the cocrystallised (original) ligands as controls into the active sites of DHFR and TNF-α. The resulting score energies were −7.93 kcal/mol and −8.19 kcal/mol, respectively, with small root mean square deviations (RMSDs) of 0.98 Å for TNF-α and 0.62 Å for DHFR (Table 5). An RMSD value of ≤ 2.0 Å is typically considered acceptable in the literature [45,46,47,48,49]. Based on these results, we applied the same docking parameters to dock eurycomanone and eurycomalactone.

Supplementary Figure S1a,b provide valuable insights into the interactions between cocrystallised ligands and the active binding sites of TNF-α and DHFR. In Figure S1a, the original ligand complexed into the active binding site of TNF-α (PDB ID: 2AZ5) is observed to form a single hydrogen bond with the tyrosine residue TYR151 at a distance of 2.45 Å. In addition, the ligand formed pi–sigma and pi–alkyl interactions with TYR119 and TYR59, which are known to be important in protein–ligand recognition and may contribute to the inhibitory activity of the compound. These findings are consistent with those reported in [30]. In Supplementary Figure S1b, the original cocrystallised ligand in PDB ID: 2AZ5 adopted a bent conformation and formed four hydrogen bonds (H-bonds) with ILE7 (3.09 Å), GLU30 (2.59 Å), ASN64 (3.44 Å),and VAL115 (3.15 Å), with relatively weak to moderate interactions. The inhibitor also established hydrophobic contacts with several active site residues of DHFR, including ALA9, LEU22, PHE31, PHE34 and ILE60. These interactions likely contributed to the inhibitory activity of the cocrystallised ligands. A comprehensive understanding of these interactions can shed light on the mechanism of action of TNF-α and DHFR inhibitors and facilitate the development of more effective drugs targeting these enzymes.

Table 5 presents the free binding energies of eurycomanone and eurycomalactone, compared to the cocrystallised ligand, within the active binding site of TNF-α (PDB ID: 2AZ5). Remarkably, the calculated binding free energies for eurycomanone and eurycomalactone are −8.83 and −7.51 kcal/mol, respectively, which are substantially similar to that of the cocrystallised ligand. Figure 5a,b show detailed 2D and 3D molecular interaction analyses of eurycomanone and eurycomalactone with the active site of TNF-α. Eurycomanone was found to form three hydrogen bonds, with two of them formed between the hydroxyl group, with an S configuration at its chiral carbon atom, and carbonyl group of the 6-hydroxy-3-methylcyclohex-2-en-1-one ring with GLY121(A) at a distance of 2.24 Å and TYR151(B) at a distance of 1.92 Å. The third hydrogen bond was formed between the hydroxyl group, with an R configuration at its chiral carbon atom, of the 2-methylenecyclohex-3-en-1-ol ring and LEU120(A) at a distance of 2.02 Å. Eurycomanone also engaged in pi–alkyl interactions with TYR59(A), TYR119(A) and TYR119(B) (See Figure 5a). In the same pattern, eurycomalactone interacted with the same residues through pi–alkyl interactions with TYR59(B), TYR119(B) and TYR151(B), and its carbonyl group of the 6-hydroxy-3-methylcyclohex-2-en-1-one ring formed a single hydrogen bond interaction with TYR119(A) at a distance of 2.01 Å. These detailed molecular interaction patterns and the Ki values suggest that both eurycomanone and eurycomalactone may serve as promising hit candidates against TNF-alpha, with their strong binding affinity likely translating into a potent inhibitory effect on TNF-α activity.

In the active binding site of DHFR (PDB ID: 5HQY), both eurycomanone and eurycomalactone exhibited free binding energies similar to the cocrystallised ligand. The calculated free binding energies for eurycomanone and eurycomalactone, which are −8.05 and −8.87 kcal/mol, respectively, suggest a promising potential for these compounds to bind to DHFR within its active binding site (Table 5).

Figure 5c,d illustrate the 2D and 3D molecular interactions of eurycomanone and eurycomalactone with DHFR. Eurycomanone is observed to establish two intermolecular hydrogen bonds with crucial residues in the active binding site of the enzyme (Figure 5c). The first hydrogen bond was formed between the carbonyl group of the 3-hydroxytetrahydro-2H-pyran-2-one ring and the guanidine group of ARG70 at a distance of 2.56 Å, while the second hydrogen bond was formed between the hydroxyl group, with an R configuration at its chiral carbon atom, of the 3-hydroxytetrahydro-2H-pyran-2-one at a distance of 1.97 Å. In the case of eurycomalactone, the 6-hydroxy-4-methylcyclohex-2-en-1-one also formed two intermolecular hydrogen bonds with TRP24 at a distance of 2.47 Å and with GLU30, with an S configuration at its chiral carbon atom, at a distance of 1.91 Å (Figure 5d). Along with these hydrogen bonds, both eurycomanone and eurycomalactone were stabilised in the active site of DHFR through hydrophobic interactions with several residues, including ILE7, ALA9, LEU22, TRP24, PHE31, PHE34 and ILE60 for eurycomanone and VAL8, ILE16, LEU22, PHE34 and VAL115 for eurycomalactone. These interactions bore a substantial resemblance to those observed with the original cocrystallised ligand, indicating that eurycomanone and eurycomalactone may inhibit DHFR through similar mechanisms or contribute to the inhibitory activity of the compounds.

From these molecular docking analyses, eurycomanone and eurycomalactone had the potential to inhibit both TNF-α and DHFR. TNF-α and DHFR also had been reported to be involved in inhibiting cell proliferations via apoptosis by others, making them among the potential targets for the treatment of cancer and other diseases [13,14,18,19]. Some anticancer drugs had been developed and targeted these TNF-α and DHFR as part of their mechanism in killing the cancer cells. For example, a few anticancer drugs already approved by the FDA and others undergoing clinical trials, include methotrexate [50,51], pemetrexed [52] and pyrimethamine [53], whereas TNF-α is a cytokine involved in inflammation and targeted for the treatment of autoimmune, inflammatory disorders [11,19] and cancer [54]. A few anticancer drugs that targeted TNF-α include doxorubicin [55], melphalan [54] and pembrolizumab [56]. To the best of our knowledge, this is the first time that eurycomanone and eurycomalactone were found to target TNF-α and DHFR via this molecular docking analysis. Nevertheless, further investigations shall be conducted to validate the inhibitory effects of eurycomanone and eurycomalactone against TNF-α and DHFR.

### 2.5. Lipinski’s Rule and ADMET of Eurycomanone and Eurycomalactone

It is known that Lipinski’s Rule of Five (RoF) is utilised to evaluate the potential of a drug to be orally bioavailable [57]. The assessment relies on molecular properties, including molecular weight, the number of hydrogen bond donors and acceptors and lipophilicity [57]. It has become essential to predict the pharmacokinetic properties of a lead compound to assess its druggable potential before entering the drug development phase [58,59]. In this study, we assessed eurycomanone, eurycomalactone and methotrexate (control) using the ADMETlab 2.0 web service tool to evaluate RoF and their pharmacokinetic properties [60]. The results of the predicted values for eurycomanone, eurycomalactone and methotrexate are presented in Table 6 and Table 7.

According to RoF, a compound is more likely to be orally active if it has no more than one violation of the following criteria: Log P is less than 5; molecular weight is less than 500 Da; hydrogen bond donor is less than 5; and hydrogen bond acceptor is less than 10 [57]. In Table 6, the predicted values of these properties for three compounds, eurycomanone, eurycomalactone and methotrexate, are presented. Eurycomanone has a molecular weight of 408.14 g/mol, 9 hydrogen bond acceptors, 4 hydrogen bond donors, and a partition coefficient (logP) of 0.215. Eurycomalactone has a molecular weight of 348.16 g/mol, 6 hydrogen bond acceptors, 1 hydrogen bond donor, and a logP of 0.655. Both compounds satisfy Lipinski’s Rule of Five, as their molecular weights are less than 500, their logP values are within the acceptable range of 0–3, and their hydrogen bond acceptor and donor counts are less than 10 and 5, respectively.

On the other hand, methotrexate has a molecular weight of 454.17 g/mol, 13 hydrogen bond acceptors, 7 hydrogen bond donors, and a logP of −2.747. Methotrexate violates Lipinski’s Rule of Five since it has more than 10 hydrogen bond acceptors and more than 5 hydrogen bond donors. Therefore, methotrexate might have poor oral bioavailability. Lipinski’s Rule of Five predicted that eurycomanone and eurycomalactone are orally bioavailable, while methotrexate might have poor oral bioavailability due to its molecular properties. However, it should be acknowledged that this rule alone is not sufficient to guarantee the efficacy or safety of a drug, as it overlooks other factors that may affect the pharmacokinetic and pharmacodynamic profiles of a compound.

To address these limitations, it is necessary to complement the Rule of Five with more comprehensive ADMET evaluations that evaluate the absorption, distribution, metabolism, excretion, and toxicity of drug candidates. In this study, we utilized the ADMETlab 2.0 web service tool to evaluate the pharmacokinetic properties of the compounds under investigation, including eurycomanone, eurycomalactone and methotrexate (See Table 7), which were also analyzed using Lipinski’s Rule of Five.

By combining the results of the Lipinski rule with the ADMET evaluation, we can obtain a more accurate and reliable prediction of the drug-likeness, pharmacokinetics and safety of the compounds, which is crucial for guiding the drug discovery and development process. Therefore, the combination of these two methods is highly recommended for identifying promising drug candidates with optimal pharmacokinetic properties, and this approach has the potential to improve the efficiency and success rate of the drug development process.

Caco-2 permeability is an important parameter when determining oral absorption and permeability in the early stages of drug development, and the ideal value is greater than −5.15 cm/s [48,61]. Eurycomanone and methotrexate showed low permeability, while eurycomalactone displayed higher permeability compared to the rest. An in vivo test on bioavailability studies had reported that eurycomanone is poorly bioavailable when given orally [62], which supported this in silico finding, whereas methotrexate is reported to be consumed either orally or intravenously [63,64]. To the best of our knowledge, there are no in vitro or in vivo studies being reported on pharmacokinetic properties on eurycomalactone. Eurycomanone, eurycomalactone and methotrexate all showed high levels of human intestinal absorption satisfaction (HIA% with a value of 30%), which is a crucial parameter associated with human intestinal absorption [48,65], affecting the way compounds pass through biological membranes under the influence of physicochemical properties. In the body, plasma protein binding plays a significant role in the dynamics of compounds [60,66]. This phenomenon is known as plasma protein binding percent (PPB%). The predicted data suggest a low protein binding potential for eurycomanone and eurycomalactone compared to the reference value of 90%. Drugs with low protein binding may have a high therapeutic index [60]. Another well-known parameter is the Blood–Brain Barrier (BBB), which facilitates the selective transfer of drug molecules between the blood and the brain parenchyma [48,60,67]. As predicted, eurycomanone and eurycomalactone were not able to cross the BBB as compared to methotrexate, which adds to the safety profile of these compounds.

A drug’s fate can be determined by its metabolism, which is characterized by the enzymatic modification or degradation of its molecules according to its therapeutic response [68]. Cytochrome P450 (CYP) enzymes are essential for the metabolism of drugs. Among 57 functional CYPs, the isoforms belonging to CYP1, CYP2 and CYP3 are responsible for the metabolism of 80% of clinical drugs [69]. Table 7 shows that eurycomanone, eurycomalactone and methotrexate showed no inhibition on CYP metabolism on other drug. An in vitro assay on eurycomanone tested on these cytochromes also had shown that there was no inhibition [70], thus indicating that eurycomanone may not interact with other drugs. The information on half-life and clearance of a drug candidate has important implications for preparing dosing regimens clinically. Half-life is defined as the time required for the concentration of a drug (typically in blood or plasma) to reduce to half of its initial value when the concentrations of the drug are in simple exponential (log−linear) decline [71]. Short half-time periods and low clearance rates were predicted from our study for eurycomanone, eurycomalactone, and methotrexate. As a leading cause of drug withdrawals from the market, drug-induced toxicity will remain a key concern for the development of novel molecules [46,48,72]. Eurycomanone and eurycomalactone showed low risk in exerting hepatotoxicity and carcinogenicity effects, whereas eurycomalactone had lower risk in exerting mutagenicity effect as compared to eurycomanone. Overall, eurycomanone and eurycomalactone showed good drug-likeness and ADMET properties; however, further in vitro and in vivo studies need to be conducted to validate these predictions.

## 3. Materials and Methods

### 3.1. Isolation and Characterisation of Eurycomanone and Eurycomalactone

The dried roots of *E. longifolia* (900 g) was extracted in ethanol (9 L) using soxhlet extraction at 55–60 °C for 18 h. The ethanol solution of extract was filtered and evaporated to yield the ethanol extract (46 g, 5.1%). The ethanol extract was fractionated using vacuum liquid chromatography on silica gel 60 (230–400 mesh) using n-hexane, n-hexane-ethyl acetate (EtOAc) (4:1, 3:2, 2:3 and 1:4) as mobile phase to obtain four fractions (FR1-FR4). A combined two fractions (FR1-FR2) (1.57 g, 0.17%) were refractionated by column chromatography (cc) on silica gel 60 (70–230 mesh) and eluted with n-hexane-EtOAc (9:1, 3:2, 2:3 and 1:9), yielding four subfractions (fr1-fr4). Subfractions fr2 (1.3 g) and fr3 (0.33 g) were then rechromatographed by cc to afford eurycomalactone (2) (7.5 mg, 0.001%) and eurycomanone (**1**) (25.1 mg, 0.002%), respectively.

### 3.2. Cell Culture and Treatments

The cancer cell lines used for this study were ovarian cancer (A2780), cervical cancer (HeLa) and colorectal cancer (HT29) as well as normal cardiomyocyte (H9C2) and liver (WRL-68) cell lines. All cell lines were purchased from American Type Culture Collections except A2780, which were purchased from the European Collection of Authenticated Cell Cultures. These cells were subcultured in Dulbecco’s Modified Eagle’s medium (Sigma-Aldrich, St. Louis, MO, USA) and supplemented with 10% fetal bovine serum (Sigma, USA), 1% penicillin–streptomycin (Sigma, USA), 1% amphotericin B (Sigma, USA) and 1% gentamicin (Sigma, USA). The cells were seeded in each well of 96 well plates and incubated in a humidified incubator at 37 °C and 5% carbon dioxide in air for 24 h. Each cell line was then treated with eurycomanone and eurycomalactone at five different concentrations (0.08, 0.4, 2, 10 and 50 µM) in triplicate. Cisplatin and methotrexate (Sigma, USA), known chemo-drugs, were also treated on these cell lines at five different concentrations (0.08, 0.4, 2, 10, 50 µM for cisplatin, 0.0008, 0.004, 0.02, 0.1, 0.5 µM for methotrexate) for the comparative studies. The treated cells were then incubated in the same incubator with the mentioned conditions for 72 h. The experiment was repeated at least three times.

### 3.3. Cells Viability Assay

Sulforhodamine B (SRB) assay [73,74] had been performed after the treated cells were incubated for 72 h. Briefly, 50 μL of ice cold tricholoroacetic acid (TCA) was added to each well and allowed to stand for 30 min at room temperature, followed by rinsing each well with tap water. Then, 100 μL of 0.4% SRB was added to each well to stain living cells for 30 min followed by a rinse with 1% acetic acid. Finally, 100 μL of Tris buffer was added to each well and the optical density (OD) of the treated and nontreated cells were read at 492 nm with a Magellan V.4 microtiter plate reader (Tecan, Salzburg, Austria). The percentage of cell viability was calculated based on (OD_492nm_ of the treated cells/OD_492nm_ of the nontreated cells) × 100. The IC_50_ values were determined from the dose–response curve of percentage of cell viability versus the concentration of the compounds (μM). Cells viability assay for each treatment was performed in triplicate in at least three independent experiments, and the IC_50_ values are given as the mean ± SEM.

### 3.4. Apoptotic Hoechst 33342 Assay

A2780, HT-29 and HeLa cancer cell lines were chosen for evaluating the apoptotic effects of eurycomanone and eurycomalactone. The morphological changes of cells undergoing apoptosis were visualised under a fluoresecence microscope after performing the Hoechst 33342 assay. Briefly, 10 × 10^4^ cells were seeded for 500 µL/well of four Labtek^®^ Chamber Slides (Thermo Fisher Scientific, Waltham, MA, USA) and incubated in 5% carbon dioxide in air for 24 h for the cells’ attachment. The cells were treated with concentrations of IC_50_/5, IC_50_ and IC_50_ × 5 values of eurycomanone and eurycomalactone in different wells and further incubated for 6, 24 and 48 h, respectively. Nontreated cells were also included in the experimental design, which acted as a negative control in this study. Following this, the media from each well were discarded and cells were fixed with 4% (*w*/*v*) paraformaldehyde for 30 min. The cells were then washed with cold phosphate buffer solution (PBS) prior to staining using Hoechst 33342, according to the method of [26]. Quantitative assessment of apoptotic cells were determined by counting apoptotic nuclei from five randomly chosen areas corroborating visual impression based on the morphology shown in Figure 3 and Figure 4. The apoptotic index (number of apoptotic nuclei per total nuclei × 100) was expressed as a percentage of the mean ± S.E.M and analysed using ANOVA followed by Tukey’s multiple comparison test.

### 3.5. Molecular Docking Simulation and ADMET Predictions

The Protein Data Bank database [75] was used to retrieve the human crystal structures of dihydrofolate reductase (DHFR) (PDB ID: 5HQY) [76] and tumor necrosis factor alpha (TNF-α) (PDB ID: 2AZ5) [77]. All water molecules and heteroatoms have been eliminated using the Biovia Discovery Studio Visualizer (San Diego, CA, USA, 2019) [78]. With MODELLER 9.18 [79], all missing residues in the crystal structures have been added and refined. Using the PDB2PQR web service (https://pdb2pqr.poissonboltzmann.org/pdb2pqr, accessed on 14 October 2022), additional treatments were performed on the crystal structures, including reconstructing missing atoms, assigning atomic charges, and radii using the SWANSON force field (AMBER ff99 charges with optimized radii) [47,48,49,80]. At pH 7.40, PROPKA3 [81], which is the most commonly used empirical pKa predictor, was used to determine the protonation states of the ionisable groups in the crystal structures. After the protonation states were assigned, the proteins were uploaded to MolProbity (http://molprobity.biochem.duke.edu/, accessed 14 October 2022) to correct bad contacts, hydrogen atom additions, and flipping of HIS, GLU, and ASN residues [47,48,49,82].

The cocrystallised ligands 5-methyl-6-[(2,3,4-trifluorophenyl)sulfanyl]thieno[2,3-d]pyrimidine-2,4-diamine) and 6,7-dimethyl-3-[[methyl-[2-[methyl-[[1-[3(trifluorome-thyl)phenyl]indol-3-yl]methyl]amino]ethyl]amino]methyl]chromen-4-one) were taken from the crystal structures of 5HQY.PDB and 2AZ5.PDB, respectively. While the chemical structures of the quassinoid derivatives were downloaded from the PubChem database [83], identification references were provided for eurycomanone (PubChem ID: 13936691) and eurycomalactone (PubChem ID: 441793). Then, the derivatives (eurycomanone and eurycomalactone) were subjected to energy minimisation using the Molecular Mechanics 2 (MM2) force field by PerkinElmer Chem3D 17.1 (PerkinElmer, Waltham, MA, USA) [46,47].

In this study, we employed molecular docking simulations to investigate the affinity of the chemical functional groups of ligands with significant amino acids for binding to the active sites of TNF-α and DHFR [84]. The inhibition constant (Ki) is a critical measure of the affinity of a ligand, such as a drug, for its target receptor. Ki is defined as the concentration of the ligand that occupies 50% of the receptors in a population [85,86,87]. Pharmacologists frequently use Ki values to assess the potency of a ligand and its potential for interacting with a receptor [85,86,87]. A lower Ki value indicates that the ligand has a higher affinity for the receptor and thus a greater potential for receptor activation or inhibition [85,86,87]. This analysis may provide insights into the molecular interactions and binding affinities of these compounds with the proteins.

AutoDockTools 1.5.6 (The Scripps Research Institute, La Jolla, CA, USA) [84]was used to add polar hydrogens and Kollman charges to the proteins, while Gasteiger charges were assigned to the cocrystallized ligands and the derivatives, and all were saved in PDBQT format. In addition, the flexibility of the ligand (active rotatable bonds) was preserved. The parameters were set as follows: the grid box size for DHFR is 40 × 40 × 40, grid spacing is 0.375, and coordinates are *x* = −4.39, *y* = 17.11, and *z* = 25.02. TNF-α has a grid box size of 40 × 40 × 40, a grid spacing of 0.375, and coordinates of *x* = −19.16, *y* = 74.45, and *z* = 33.83. These coordinates were centered on the active binding sites and were saved in grid parameter files (GPFs). Proteins were set as rigid, while ligands were set as flexible. The number of genetic algorithm runs was 100, the number of population sizes was 150, the maximum number of evaluations was 2,500,000 (medium) and the maximum number of generations was 27,000. The Lamarckian genetic algorithm was selected to accomplish this process, and the remaining parameters were retained as defaults and saved in docking parameter files (DPFs). The docking processes were simulated using AutoDock 4.2 [88]. The Biovia Discovery Studio Visualizer [78], which allows for both 2D and 3D visualization, was used to visualize the molecular interactions between the ligands and the active site of proteins.

ADMETlab 2.0 web service tool was used to predict the drug-likeness via Lipinski’s rule of five (RoF) and pharmacokinetic properties (https://admetmesh.scbdd.com/service/evaluation/cal (accessed on 28 October 2022)), which anticipates mutagenicity (Ames test), carcinogenicity and permeability of the BBB, as well as absorption of human intestinal protein and plasma protein binding [60]. The two-dimensional chemical structures of eurycomanone and eurycomalactone were converted to the SMILES format and submitted to ADMETlab 2.0 to obtain the results. Methotrexate, an anticancer drug with antifolate property, was included in this pharmacokinetic analysis for comparison studies.

## 4. Conclusions

In conclusion, both eurycomanone and eurycomalactone showed strong in vitro anticancer activities and their mechanisms of action was via apoptosis in killing the cancer cells. The molecular docking study showed that both compounds can target DHFR and TNF-α by binding to their active sites and forming hydrogen bonds with the key residues. The compounds also exhibited hydrophobic interactions that enhanced their stability. In addition, eurycomanone and eurycomalactone may be suitable to be developed as drug candidates because they both obeyed Lipinski’s rule. These two compounds are also predicted to be safe, implying that these compounds could serve as direct inhibitors against the target proteins. However, further molecular dynamics, in vitro and immuno-assays should be conducted to confirm the predicted values.

## Figures and Tables

**Table 1:** ^1^H and ^13^C NMR spectral data of eurycomanone (**1**) and eurycomalactone (**2**) (400, 100 MHz in CDCl_3_).

Eurycomanone (1)			Eurycomalactone (2)		
No	^1^H δ ppm	^13^C δ ppm	No	^1^H δ ppm	^13^C δ ppm
1	4.03 (1H, d, 8 Hz)	81.40	1	4.05 (1H, s)	81.27
2	-	197.89	2	-	197.41
3	6.14 (1H, d, 1.5 Hz)	162.98	3	6.13 (1H, s)	124.39
4	-	126.48	4	-	162.17
5	2.36 (1H, td, 2.4 Hz)	48.14	5	2.91 (1H, m)	49.38
6	2.08 (2H, m)	42.58	6	2.79 (2H, m)	36.21
7	-	199.32	7	-	205.56
8	-	53.02	8	-	51.16
9	2.02 (1H, t, 2.7, 13.3 Hz)	50.42	9	1.88 (1H, d, 3.2 Hz)	49.06
10	-	46.34	10	-	46.90
11	4.53 (1H, t, 6.8 Hz)	68.10	11	4.78 (1H, t, 4 Hz)	69.75
12	4.59 (1H, d, 8 Hz)	72.21	12	4.39 (1H, d, 4.4 Hz)	83.16
13	-	119.80	13	2.89 (1H, m)	32.33
14	3.25 (1H,d, 12.5 Hz)	76.28	14	3.02 (1H, m)	52.88
15 16	5.25 (1H,t, 2.5, 18.7 Hz) -	79.81 174.35	15	-	176.31
4-CH_3_	1.81 (3H, s)	10.81	4-CH_3_	1.64 (3H, s)	23.64
10-CH_3_	2.00 (3H, s)	26.11	8-CH_3_	1.96 (3H, s)	21.97
8-CH_2_	2.07 (2H, m)	84.95	10-CH_3_	1.27 (3H, s)	12.19
13′	7.6 (2H,s)	108.71	13-CH_3_	1.18 (3H, d, 6.4 Hz)	32.33

**Table 2:** The IC_50_ values (μM) ± SEM (*n* = 9) of eurycomanone, eurycomalactone, cisplatin and methotrexate tested in ovarian (A2780), cervical (HeLa) and colorectal cancer (HT29) cancer cell lines, and cardiomyocyte (H9C2) and liver (WRL-68) normal cell lines.

Compound	A2780	HeLa	HT-29	H9C2	WRL-68
Eurycomanone	1.37 ± 0.13	4.58 ± 0.090	1.22 ± 0.11	>50	1.34 ± 0.046
Eurycomalactone	2.46 ± 0.081	1.60 ± 0.12	2.21 ± 0.049	7.00 ± 0.43	2.71 ± 0.042
Cisplatin	1.77 ± 0.018	1.54 ± 0.12	1.38 ± 0.037	14.07 ± 1.14	1.13 ± 0.098
Methotrexate	0.016 ± 0.00050	0.094 ± 0.0043	0.059 ± 0.0010	>50	0.015 ± 0.00041

Note: SEM (Standard Error of the Mean) < 5%. Compound with IC_50_ values less than 50 µM is considered active [39].

**Table 3:** Apoptotic Indices of A2780, HT-29 and HeLa when being treated with eurycomanone.

Concentrations/ Incubation Time	IC_50_/5	IC_50_	IC_50_ × 5
Cell Line: A2780			
6 h	4.30 ± 0.34 ^a/x^	8.48 ± 0.60 ^ab/x^	13.01 ± 0.29 ^ac,bc/x^
24 h	7.11 ± 1.60 ^a/xy^	13.13 ± 1.30 ^ab/y^	28.90 ± 0.93 ^ac,bc/xy^
48 h	14.72 ± 0.59 ^a/xz,yz^	31.74 ± 3.19 ^ab/xz,yz^	100.00 ± 0.00 ^ac,bc/xz,yz^
Cell Line: HT-29			
6 h	4.08 ± 0.81 ^a/x^	5.24 ± 0.17 ^b/x^	5.97 ± 0.35 ^ac/x^
24 h	4.18 ± 0.21 ^a/y^	6.30 ± 1.01 ^b/y^	10.95 ± 0.71 ^ac,bc/y^
48 h	4.24 ± 0.20 ^a/z^	14.80 ± 0.56 ^ab/xz,yz^	26.20 ± 1.48 ^ac,bc/xz,yz^
Cell Line: HeLa			
6 h	8.42 ± 0.20 ^a/x^	19.98 ± 0.76 ^ab/x^	41.37 ± 0.24 ^ac,bc/x^
24 h	13.76 ± 1.26 ^a/xy^	52.57 ± 1.40 ^ab/y^	94.56 ± 1.16 ^ac,bc/xy^
48 h	16.79 ± 0.92 ^a/xz,yz^	61.16 ± 0.63 ^ab/xz,yz^	100.00 ± 0.00 ^ac,bc/xz,yz^

Statistical analysis was performed using one-way analysis of variance (ANOVA) followed by Tukey’s multiple comparison test using Graphpad Prism version 7. ^a,b,c^ letters represent significant differences within the groups when compared to IC_50_/5 (dose-dependent experiment) while ^x,y,z^ represent significant differences between the groups when compared to 6 h of treatment (time-dependent experiment).

**Table 4:** Percentage of apoptotic indices of A2780, HT-29 and HeLa when being treated with eurycomalactone.

Concentrations/ Incubation Time (h)	IC_50_/5	IC_50_	IC_50_ × 5
Cell Line: A2780			
6 h	3.84 ± 0.10 ^a/x^	4.26 ± 0.64 ^b/x^	15.81 ± 0.38 ^ac,bc/x^
24 h	4.12 ± 0.16 ^a/y^	6.29 ± 0.21 ^ab/y^	Cells died and completely detached ^ac,bc/xy^
48 h	5.16 ± 0.065 ^a/xz,yz^	7.93 ± 2.28 ^b/xz^	Cells died and completely detached ^ac,bc/xz^
Cell Line: HT-29			
6 h	3.79 ± 0.45 ^a/x^	4.51 ± 0.23 ^b/x^	6.52 ± 1.22 ^ac,bc/x^
24 h	7.71 ± 0.51 ^a/xy^	12.41 ± 0.77 ^ab/xy^	20.21 ± 1.52 ^ac,bc/xy^
48 h	8.86 ± 0.68 ^a/xz^	14.28 ± 0.84 ^ab/xz,yz^	100.00 ± 0.00 ^ac,bc/xz,yz^
Cell Line: HeLa			
6 h	5.45 ± 0.23 ^a/x^	17.67 ± 0.77 ^ab/x^	31.87 ± 2.19 ^ac,bc/x^
24 h	8.94 ± 0.21 ^a/xy^	32.00 ± 1.57 ^ab/xy^	62.20 ± 1.35 ^ac,bc/xy^
48 h	14.78 ± 0.12 ^a/xz,yz^	36.71 ± 1.19 ^ab/xz,yz^	100.00 ± 0.00 ^ac,bc/xz,yz^

Statistical analysis was performed using one-way analysis of variance (ANOVA) followed by Tukey’s multiple comparison test using Graphpad Prism version 7. ^a,b,c^ letters represent significant differences within the groups when compared to IC_50_/5 (dose-dependent experiment) while ^x,y,z^ represent significant differences between the groups when compared to 6 h of treatment (time-dependent experiment).

**Table 5:** The free binding energies (ΔGbind) and inhibition constants (Ki) for eurycomanone, eurycomalactone and the cocrystallised ligands of TNF-α (PDB ID: 2AZ5) and DHFR (PDB ID: 5HQY) were calculated into the active binding sites using AutoDock 4.2.

Compound	TNF-α		DHFR	
	*ΔG_bind_ (kcal/mol)	*K_i_ (Micromolar uM)	*ΔG_bind_ (kcal/mol)	*K_i_ (Micromolar uM)
Eurycomanone	−8.83	0.34	−8.05	1.25
Eurycomalactone	−7.51	3.11	−8.87	0.32
*124037103	*****	*****	−8.19	0.99
*5327044	−7.93	1.53	*****	*****

*ΔG_bind_: Gibbs free binding energy (kcal/mol), *Ki: Inhibition constant (uM). *124037103: The PubChem ID of the cocrystallised ligands in DHFR (PDB ID: 5HQY). *5327044: The PubChem ID of the cocrystallised ligands in TNF-α (PDB ID: 2AZ5). ***** means not relevant.

**Table 6:** *Lipinski’s Rule of Five (RoF) predicted values for eurycomanone, eurycomalactone and methotrexate.

Compounds	*M.W (g/mol)	*Hacc	*Hdon	*logP
Eurycomanone	408.14	9	4	0.215
Eurycomalactone	348.16	6	1	0.655
Methotrexate	454.17	13	7	−2.747

*M.W: Molecular weight (g/mol), *logP: Partition coefficient (Lipophilicity), *Hacc: Hydrogen bond acceptor, *Hdon: Hydrogen bond donor. *Lipinski Rule: M.W ≤ 500; logP ≤ 5; Hacc ≤ 10; Hdon ≤ 5, an orally active drug has no more than one violation of these criteria.

**Table 7:** Predicted ADMET properties of eurycomanone, eurycomalactone and methotrexate using ADMETlab 2.0.

Property	Model Name	Predicted Value			Comment
		Eurycomanone	Eurycomalactone	Methotrexate	
Absorption	Papp (Caco-2 Permeability) cm/s	−5.54	−5.01	−6.73	* Papp ideal value is > −5.15 cm/s
	HIA (Human Intestinal Absorption)%	5.78	4.01	3.70	* HIA idea value is < 30%
Distribution	*PPB (Plasma Protein Binding)%	52.15	52.66	55.23	* PPB ideal value is < 90%
	Cross BBB (Blood Brain Barrier)	No	Yes	No	
Metabolism	CYP1A2 substrate	No	No	No	
	CYP2C19 substrate	No	No	No	
	CYP2C9 substrate	No	No	No	
	CYP2D6 substrate	No	No	No	
	CYP1A2 inhibitor	No	No	No	
	CYP2C19 inhibitor	No	No	No	
	CYP2C9 inhibitor	No	No	No	
	CYP3A4 inhibitor	No	No	No	
Excretion	*CL (Clearance Rate) mL/min/kg	1.75	2.31	2.52	High: CL >15 mL/min/kg Moderate: CL 5–15 mL/min/kg Low: CL <5 mL/min/kg
	T ½ (Half Lifetime) hr	0.03	0.18	0.89	Long half-life: >3 h Short half-life: <3 h
Toxicity	H-HT (Human Hepatotoxicity)	+	+	+++	+ Low risk to be toxic. ++ Moderate risk to be toxic. +++ High risk to be toxic.
	AMES (Ames Mutagenicity)	++	+	+	
	Carcinogenicity	+	+	++	

*PBB: Plasma Protein Binding (PPB) Optimal: <90%. High protein-bound drugs may have a low therapeutic index. *CL (Clearance Rate) mL/min/kg: high: CL >15 mL/min/kg, moderate: CL 5–15 mL/min/kg, and low: CL <5 mL/min/kg.

## Data Availability

All relevant data already included in the text.

## Supplementary Materials

The following supporting information can be downloaded at: https://www.mdpi.com/article/10.3390/plants12152827/s1, Figure S1: The binding interactions of the cocrystallized ligands in the active binding sites of TNF-α (PDB ID: 2AZ5) and DHFR (PDB ID: 5HQY).
