# Supplementary material for: In Vitro and In Silico Analysis of the Anticancer Effects of Eurycomanone and Eurycomalactone from Eurycoma longifolia
Source: Plants (Basel). 2023 Jul 31;12(15):2827. doi: 10.3390/plants12152827 (PMC10421158; doi:10.3390/plants12152827)
Supplement: Supplementary file 1 [file plants-12-02827-s001.zip › plants-2425061-supplementary.pdf]

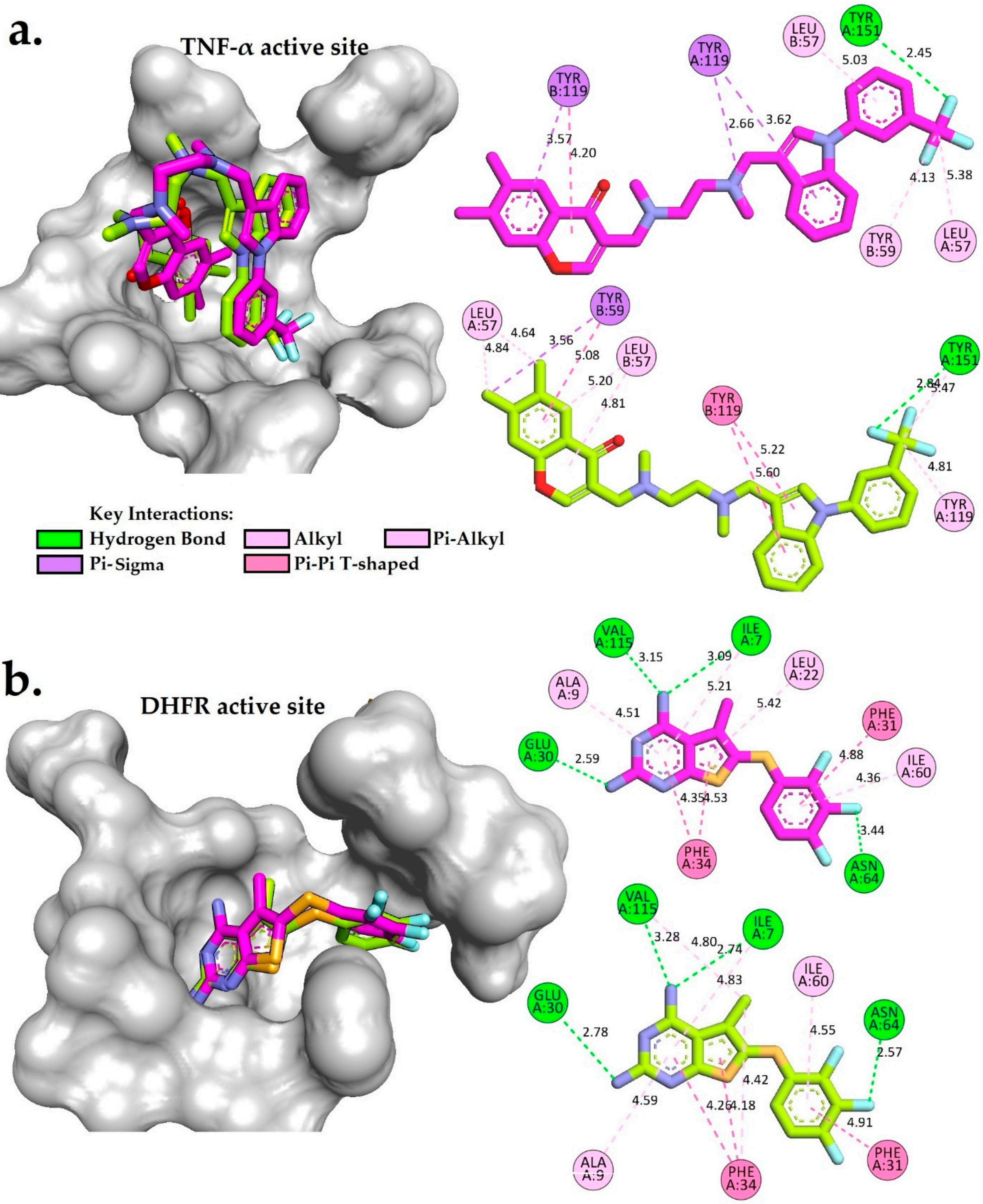

Figure S1. The binding interactions of the cocrystallized ligands in the active binding sites of TNF-α (PDB ID: 2AZ5) and DHFR (PDB ID: 5HQY).

Note: a) Superimposition of the original crystal structure (C pink, O red, F cyan, S orange, and N blue) and docked model (C lime, O red, F cyan, S orange, and N blue) into the active binding site of the Tumor Necrosis Factor Alpha (TNF-α) crystal structure (PDB ID: 2AZ5) and their 2D interactions (RMSD = 0.98 Å). b) Superimposition of the original crystal structure (C pink, O red, F cyan, S orange, and N blue) and the docked model (C lime, O red, F cyan, S orange, and N blue) into the active binding site of dihydrofolate reductase (DHFR) crystal structure (PDB ID: 5HQY) with RMSD = 0.62 Å and their 2D interactions. Some regions of the protein are omitted to facilitate visualisation. These models were generated using BIOVIA Discovery Studio Visualizer.
